# Risk factors among latin american women with drug abuse

**DOI:** 10.1192/j.eurpsy.2026.11932

**Published:** 2026-07-31

**Authors:** V. C. Valdez, A. C. Risco Solis, S. S. Lopez Flores, R. S. Armijos Nuñez, A. E. Martínez Salazar

**Affiliations:** Facultad de Ciencias de la Salud, Universidad Católica de Santiago de Guayaquil, Guayaquil, Ecuador

## Abstract

**Introduction:**

In the last decade it has been established an increase in drug abuse among women,(NIDA) Factors and circumstances that lead women to develop this illness are many, well explore certain elements in this research.

**Objectives:**

The aim of this study is to identify the main risk factors associated with substance use and abuse in Latin-American women.

**Methods:**

An observational, cross-sectional and descriptive study was conducted at the Gynecology Department of the Hospital General del Norte of Guayaquil during August 2025. The total sample was 95. An Inform consent procedure was notified to each women at the gynecological area. The instruments used to collect the data included a structured admission form, the Alcohol Use Disorders Identification Test (AUDIT), the Drug Use Disorders Identification Test (DUDIT), the Emotional Dependence Scale(EDS), and a Violence Form.

**Results:**

Among the participants, 63.13% reported experiencing some type of violence, predominantly psychological (80%), followed by physical (46.67%), financial (31.67%), and sexual (18.33%). In 22% of cases, polyvictimization was reported. AUDIT results showed that 44.74% of women who consumed alcohol were in the high-risk or probable addiction categories. DUDIT results showed that 27% of drug users were likely to be addicted. 51.67% of the participants displayed moderate levels of emotional dependence, while 18.33% showed high levels.

**Conclusions:**

The study revealed a strong association between violence, emotional dependence and substance use among Latin-American women. The elevated prevalence of high-risk substance consumption in this study highlights the urgent need for comprehensive, multidisciplinary, intervention strategies. These strategies should integrate substance abuse treatment with addressing gender-based violence and psychological vulnerability.

**Disclosure of Interest:**

None Declared

